# Peripartum Cardiomyopathy in the Obstetric Intensive Care Unit: Seasonal Associations and the Impact of Winter in the Kashmir Valley

**DOI:** 10.7759/cureus.116048

**Published:** 2026-09-10

**Authors:** Eman A Khan, Farhana Giri, Adnan Qadri, Shahira Wani

**Affiliations:** 1 Anesthesiology, Amandeep Hospital, Srinagar, IND; 2 Obstetrics and Gynecology, Government Medical College Doda, Doda, IND; 3 Anesthesiology, Paras Hospital, Srinagar, IND; 4 Obstetrics and Gynecology, Government Medical College Srinagar, Srinagar, IND

**Keywords:** heart failure in pregnancy, kashmir valley, maternal mortality, obstetric intensive care, peripartum cardiomyopathy, seasonal variation, winter

## Abstract

Background

Peripartum cardiomyopathy (PPCM) is a form of idiopathic systolic heart failure that presents in late pregnancy or the early postpartum period in the absence of an identifiable etiology. Because its symptoms overlap with those of normal pregnancy, the diagnosis is frequently delayed or missed. Seasonal peaks in cardiovascular morbidity and mortality are well described in older, non-obstetric populations, but no previous study has examined this phenomenon in obstetric patients with PPCM. We aimed to determine whether the proportion of obstetric intensive care unit (ICU) admissions attributable to PPCM and its associated mortality are higher during winter than during the remaining seasons.

Materials and methods

This retrospective observational study included all obstetric patients admitted to the ICU of a tertiary-care referral center in the Kashmir Valley, North India, between January 1, 2018 and April 1, 2024. Medical records were reviewed to identify cases of new-onset PPCM; women with pre-existing cardiac disease were excluded. Admissions were stratified by season (spring: February 15 to May 15; summer: May 15 to August 15; autumn: August 15 to November 15; winter: November 15 to February 15). Groups were compared using the chi-square test, with odds ratios (OR) and 95% confidence intervals (CI). A p-value of less than 0.05 was considered significant.

Results

Of 1,899 obstetric ICU admissions, 166 (8.74%) received a new diagnosis of PPCM. Winter accounted for 88 of the 166 PPCM admissions (53.01%), compared with 78 (46.99%) for the other three seasons combined, whereas only 365 of 1,733 non-PPCM admissions (21.06%) occurred in winter. The odds that an obstetric ICU admission was attributable to PPCM were therefore more than four times higher in winter (OR 4.22, 95% CI 3.05-5.85; χ² = 85.14; p < 0.001). Fifteen patients with PPCM died in hospital, of whom 11 (73.33%) died during winter; the case fatality rate among patients with PPCM was 12.50% in winter compared with 5.13% in the other seasons. Hypertensive disorder of pregnancy was the commonest comorbidity (52.41%), and 43.98% of patients presented in New York Heart Association functional class IV.

Conclusions

A clinically and statistically significant seasonal association was observed regarding the proportion of PPCM admissions among obstetric ICU patients in the Kashmir Valley, with the greatest burden concentrated in winter; however, these findings may be confounded by delayed presentation due to winter transport difficulties.

Heightened surveillance, intensified antenatal follow-up, and a low threshold for echocardiography during the cold season are recommended, particularly for women referred from peripheral facilities.

## Introduction

Heart failure was first described as an emerging epidemic approximately 25 years ago [1]. Despite global improvements in maternal care, pregnancy-related deaths continue to rise in several settings, and cardiovascular disease now ranks among the leading causes of maternal death and severe maternal morbidity [2]. Among critically ill obstetric patients with cardiac disease admitted to the intensive care unit (ICU), mortality has been reported to reach 50% [3]. The physiological cardiovascular adaptations of pregnancy, which include an increased cardiac output, an expanded blood volume, a raised red cell mass, and reduced hemoglobin and hematocrit concentrations, impose an additional burden on the maternal heart [4].

Peripartum cardiomyopathy (PPCM) is a form of idiopathic systolic heart failure that presents in the final weeks of pregnancy or within five months of delivery. Its exact pathogenesis remains unclear, but recognized risk factors include multiparity, pre-eclampsia and eclampsia, advanced maternal age, African ancestry, obesity, chronic hypertension, and prolonged tocolytic therapy [5]. Because the symptoms of PPCM closely mimic those of normal pregnancy, the diagnosis is frequently delayed, with potentially life-threatening consequences for both mother and fetus [5].

Seasonal peaks in cardiovascular morbidity and mortality, including stroke, myocardial infarction, and heart failure, have been documented in older, non-obstetric populations, with the greatest burden observed during the winter months [6-8]. The physiological responses to cold, namely sympathetic activation, peripheral vasoconstriction, and a consequent rise in left ventricular afterload, may overload a heart that is already stressed by pregnancy and thereby precipitate acute decompensated heart failure [6,7]. Concurrent winter respiratory infections may further predispose susceptible individuals to myocardial inflammation [8]. In the Kashmir Valley, ambient temperatures fall to -6°C to -8°C during winter, making this an especially pertinent clinical question.

While seasonal variations in PPCM admissions have been noted previously (e.g., during the harmattan and rainy seasons in Nigeria by Isezuo and Abubakar), to our knowledge, this is the first study to examine seasonal variation specifically among critically ill obstetric patients in a high-altitude, cold-winter ICU setting. For anesthesiologists and intensivists, an understanding of the temporal clustering of PPCM is clinically important, because it may facilitate early diagnosis, prompt referral and, in peripheral facilities that lack echocardiography or cardiology support, inform the decision to avoid spinal anesthesia, which can precipitate acute decompensation in unrecognized compensated heart failure. The primary aim of this study was therefore to determine whether the proportion of obstetric ICU admissions attributable to PPCM and its associated mortality are significantly higher during winter than during the other seasons in an obstetric ICU serving the Kashmir Valley.

## Materials and methods

Study setting

This retrospective observational study was conducted in the multidisciplinary ICU of a 700-bed tertiary-care teaching hospital in the Kashmir Valley, North India, which is the largest maternity hospital in the region and the designated referral center for high-risk pregnancies. In April 2022 alone, 13,827 patients attended the outpatient department, and approximately 3,000 patients were hospitalized, with a mean of 80 deliveries (spontaneous and cesarean) performed each day.

Study period and participants

The study was approved by the Institutional Research Committee of the participating hospital. All obstetric patients admitted to the ICU between January 1, 2018, and April 1, 2024, were eligible for inclusion, and the entire study period was captured retrospectively from medical records. All obstetric ICU admissions were retrieved to establish a baseline comparative cohort. From this broad group, the PPCM cases were identified by strictly excluding patients with known pre-existing cardiac disease, in order to limit ascertainment bias. Because the study was retrospective in design and all records were de-identified before analysis, the requirement for individual informed consent was waived by the ethics committee.

Case identification and definitions

Cases were identified by systematically querying the hospital's electronic medical records (EMR) and intensive care admission registers. Diagnostic codes and admission notes for heart failure, dilated cardiomyopathy, and peripartum cardiomyopathy were cross-referenced with cardiology consultation and echocardiography reports to confirm the diagnosis.

PPCM was diagnosed based on new-onset heart failure presenting in the third trimester of pregnancy or within five months of delivery, together with echocardiographic evidence of left ventricular systolic dysfunction (ejection fraction < 45%), in the absence of an identifiable alternative cause, following the Heart Failure Association of the European Society of Cardiology criteria [9]. The New York Heart Association (NYHA) Functional Classification was used to grade heart failure symptoms [10], and left ventricular ejection fraction was quantified according to the American Society of Echocardiography guidelines [11].

Data collection

The following variables were extracted from the medical record of each patient: age, area of residence, mode of admission (direct or referral), last menstrual period, antenatal or postnatal status, comorbidities, NYHA functional class, echocardiographic left ventricular ejection fraction (EF), and clinical outcome (discharge or in-hospital death). Alternative structural or chronic hypertensive causes of left ventricular dysfunction were excluded via formal cardiology consultation and review of antenatal records.

Seasonal classification

Based on the date of admission, each case was assigned to one of four three-month seasons in accordance with regional meteorological and climatological patterns for the Kashmir Valley [12]: spring (February 15 to May 15), summer (May 15 to August 15), autumn (August 15 to November 15), and winter (November 15 to February 15).

Statistical analysis

Statistical analysis was performed using SPSS Statistics (Version 26.0, IBM Corp., Armonk, NY). Categorical variables are presented as frequencies and percentages (n, %). The assumptions for the chi-square test of independence were assessed to ensure that all expected cell frequencies were greater than five. Any missing data were handled using complete case analysis. PPCM admissions were compared with non-PPCM obstetric ICU admissions, and PPCM deaths occurring in winter were compared with those occurring in the other seasons using the chi-square test. Odds ratios (OR) with 95% confidence intervals (CI) were calculated. All statistical tests were two-sided. Complete statistical results are reported, including degrees of freedom (df), exact p-values, and effect sizes measured via the Phi coefficient. A p-value of less than 0.05 was considered statistically significant. Analyses were bivariate; no multivariable model was fitted.

## Results

A total of 1,899 obstetric patients were admitted to the ICU during the study period after the exclusion of women with pre-existing cardiac disease. Of these, 166 (8.74%) received a new diagnosis of PPCM. The demographic characteristics of the cohort are summarized in Table 1.

**Table 1:** Demographic profile of admitted patients Percentages are calculated within each column group (PPCM, n = 166; non-PPCM, n = 1,733). PPCM: peripartum cardiomyopathy.

Variable	Category	PPCM cases (n)	%	Non-PPCM cases (n)	%
Age (years)	18-20	2	1.20	52	3.00
	21-30	42	25.30	379	21.87
	31-40	64	38.55	621	35.83
	41 and above	58	34.94	681	39.30
Residence	Urban	64	38.55	391	22.56
	Rural	102	61.45	1342	77.44
Mode of admission	Direct	72	43.37	558	32.20
	Referral	94	56.63	1175	67.80

Age and residence

Among patients with PPCM, the largest age group was 31 to 40 years (n = 64; 38.55%), followed by 41 years and above (n = 58; 34.94%), a distribution consistent with the recognized unadjusted association between advanced maternal age and PPCM [13]. Among patients with PPCM living in rural areas, more than half were referred from peripheral facilities (n = 94; 56.63%), reflecting limited antenatal access and higher parity among remote populations.

Clinical characteristics

The clinical characteristics of the 166 patients with PPCM are detailed in Table 2.

**Table 2:** Comparison of winter and non-winter PPCM Values are presented as n (%). P-values are based on a comparison between winter and non-winter PPCM groups. None of the assessed variables differed significantly between the two groups (p > 0.05). The total number of comorbidities may not add up, as some patients had more than 1 comorbidity. PPCM: peripartum cardiomyopathy; NYHA: New York Heart Association; LVEF: left ventricular ejection fraction

Variable	Winter PPCM (n = 88)	Non-winter PPCM (n = 78)	p-value
Comorbidities, n (%)			
Hypertensive disorder	43 (48.86)	45 (57.69)	0.326
Anemia	23 (26.14)	18 (23.08)	0.783
Gestational diabetes mellitus	14 (15.91)	15 (19.23)	0.721
Hypothyroidism	16 (18.18)	12 (15.38)	0.785
No known comorbidity	20 (22.73)	16 (20.51)	0.875
Timing of presentation, n (%)			1.000
Antepartum	43 (48.86)	39 (50.00)	
Postpartum	45 (51.14)	39 (50.00)	
NYHA functional class, n (%)			0.991
II	20 (22.73)	18 (23.08)	
III	31 (35.23)	28 (35.90)	
IV	37 (42.05)	32 (41.03)	
LVEF, n (%)			0.163
10–20%	1 (1.14)	2 (2.56)	
21–30%	20 (22.73)	8 (10.26)	
31–40%	45 (51.14)	43 (55.13)	
41–44%	22 (25.00)	25 (32.05)	

With regard to the timing of presentation, 60 patients (36.14%) were admitted between 34 and 38 weeks of gestation and 24 (14.46%) at 38 weeks of gestation or beyond, whereas 43 (25.90%) presented during the first five postpartum days and 39 (23.49%) between postpartum days 6 and 11. The most severe dyspnea category, NYHA class IV, was recorded in 73 patients (43.98%), and a further 57 patients (34.34%) were in class III. Echocardiography showed an EF of 31% to 40% in the majority of patients (n = 87; 52.41%), whereas only three patients (1.81%) had an EF as low as 10% to 20%. The most prevalent comorbidity was a hypertensive disorder of pregnancy (n = 87; 52.41%), followed by anemia (n = 36; 21.69%); 20 patients (12.05%) had no known comorbidity.

The distribution of non-PPCM ICU admissions between winter and the remaining seasons is presented in Table 3.

**Table 3:** Other causes of intensive care unit admission, excluding peripartum cardiomyopathy and gynecological cases Percentages are calculated within each seasonal column. Indented entries are subcategories of obstetric hemorrhage.

Condition	Winter (n = 365)		Other seasons (n = 1,368)	
	n	%	n	%
Obstetric hemorrhage (total)	189	51.78	703	51.39
Antepartum hemorrhage	66	18.08	245	17.91
Postpartum hemorrhage	84	23.01	310	22.66
Abortion	7	1.92	51	3.73
Ectopic pregnancy	29	7.95	89	6.51
Ruptured uterus	2	0.55	6	0.44
Molar pregnancy	1	0.27	2	0.15
Hypertensive disorder of pregnancy	105	28.77	357	26.10
Severe anemia	16	4.38	64	4.68
Sepsis	12	3.29	54	3.95
Respiratory tract infection or pneumonia	22	6.03	95	6.94
Other conditions	20	5.48	95	6.94

Seasonal variation in admissions 

Of the 166 PPCM admissions, 88 (53.01%) occurred during winter alone, compared with 78 (46.99%) across the other three seasons combined. Among the 1,733 non-PPCM admissions, 365 (21.06%) occurred in winter and 1,368 (78.94%) in the other seasons. Because total regional delivery denominators were not available, outcomes are expressed as a proportion of total obstetric ICU admissions. The odds that an obstetric ICU admission was attributable to PPCM were therefore more than four times higher in winter than in the remaining seasons (OR = 4.22, 95% CI: 3.05-5.85; χ²(1) = 85.14; p < 0.001; Phi coefficient = 0.21), indicating a marked winter preponderance of PPCM (Table 4).

**Table 4:** Comparison of peripartum cardiomyopathy admissions in winter versus the other seasons Each comparison is a two-by-two contingency analysis with one degree of freedom. CI: confidence interval; ICU: intensive care unit; PPCM: peripartum cardiomyopathy; χ²: chi-square value.

Comparison	Season	PPCM	Non-PPCM	χ²	p-value	Odds ratio (95% CI)
ICU admissions	Winter	88	365	85.14	< 0.001	4.22 (3.05-5.85)
	Other seasons	78	1,368	-	-	Reference

Fifteen patients with PPCM died in hospital. Eleven of these 15 deaths (73.33%) occurred during winter and four (26.67%) during the remaining three seasons, giving a case fatality rate among patients with PPCM of 12.50% (n = 11/88) in winter compared with 5.13% (n = 4/78) in the other seasons. Using Fisher's exact test, this difference in mortality was not statistically significant (p = 0.112)

The season-by-season distribution of PPCM admissions and deaths is presented in Table 5 and illustrated in Figure 1.

**Table 5:** Seasonal distribution of peripartum cardiomyopathy admissions and in-hospital deaths Percentages are calculated across the four seasons (admissions, n = 166; deaths, n = 15). PPCM: peripartum cardiomyopathy.

Season	PPCM admissions (n)	% of all PPCM admissions	PPCM deaths (n)	% of all PPCM deaths
Spring	24	14.46	1	6.67
Summer	22	13.25	1	6.67
Autumn	32	19.28	2	13.33
Winter	88	53.01	11	73.33
Total	166	100.00	15	100.00

**Figure 1:**
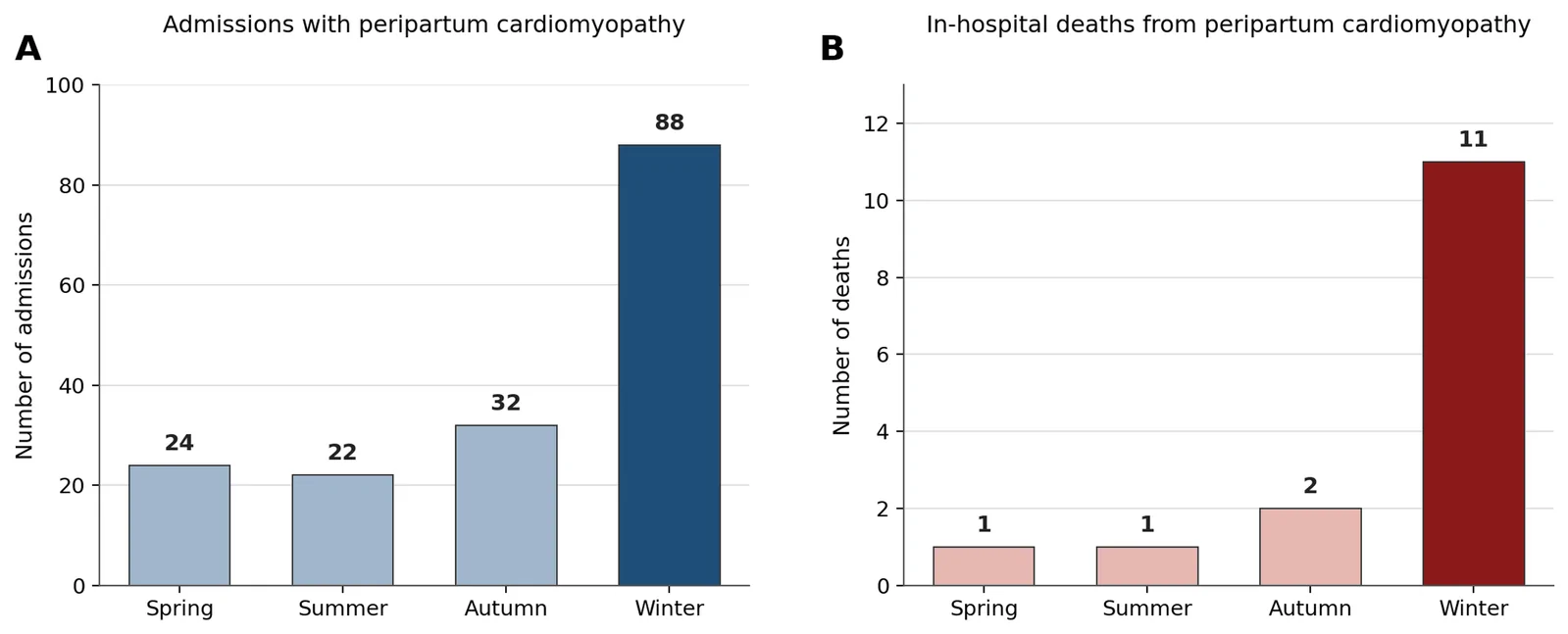
Seasonal distribution of peripartum cardiomyopathy admissions and in-hospital deaths Panel A shows the number of intensive care unit admissions with peripartum cardiomyopathy in each season; panel B shows the number of in-hospital deaths from peripartum cardiomyopathy in each season. Winter bars are shaded darker. Data are from the same cohort as Table 5.

## Discussion

To our knowledge, this is the first study to document a significant seasonal variation in the proportion of obstetric ICU admissions attributable to PPCM and mortality from PPCM specifically among obstetric patients. PPCM is a disorder of uncertain etiology that occurs in late pregnancy or the early puerperium, and its reported proportion of obstetric ICU admissions attributable to PPCM varies widely by geography and ethnicity [5]. Our findings show that winter is the season of greatest risk for both presentation with PPCM and death from it: more than half of all PPCM admissions (53.01%) and nearly three-quarters of all PPCM deaths (73.33%) were concentrated in this single season.

These observations are consistent with the published literature on seasonal cardiovascular morbidity in non-obstetric populations. Iorgoveanu et al. [5] concluded that the pathogenesis of PPCM is incompletely understood and probably multifactorial, with hypertensive disorders, advanced maternal age, and African ancestry among the most consistently identified risk factors; the first three of these were also prominent in our cohort. Izzo et al. [7] demonstrated that seasonal cold exposure raises heart rate and systemic vascular resistance while reducing cardiac output and stroke volume, a hemodynamic profile that may overload an already stressed peripartum myocardium. Peripheral vasoconstriction induced by cold can precipitate acute pulmonary edema by increasing left ventricular afterload, thereby converting compensated heart failure into decompensated heart failure [6]. This mechanism is particularly relevant in the Kashmir Valley, where winter temperatures frequently fall to -6°C to -8°C. However, severe winter weather frequently produces heavy snowfall and road blockages that delay emergency transfer, so it is difficult to isolate how much of the winter excess in mortality is driven by cold-induced increases in left ventricular afterload as opposed to delayed presentation and prolonged cardiogenic shock before arrival at the tertiary ICU. Because the analysis is unadjusted, we cannot isolate the direct effect of cold exposure from critical confounders like delayed presentation due to road blockages or differing baseline severity of comorbidities.

A further observation deserves comment. Overall in-hospital mortality among patients with PPCM in this cohort (n = 15/166; 9.04%) was not higher than that among the remaining obstetric ICU population (n = 245/1,733; 14.14%). The distinguishing feature of PPCM in our series was therefore not its absolute case fatality, which was comparable to that of other critical obstetric conditions managed in the same unit, but the pronounced seasonal concentration of both the proportion of obstetric ICU admissions attributable to PPCM and its deaths: winter accounted for 53.01% of PPCM admissions and 73.33% of PPCM deaths, a degree of clustering not seen in the other diagnostic categories.

This study emphasizes that we should keep a high index of suspicion for PPCM during the winter season and carefully assess patients with signs of dyspnea. We should exercise extreme caution with neuraxial techniques where compensated PPCM is suspected, as abrupt hemodynamic shifts can precipitate acute decompensation.

Limitations

This study has several limitations that should be considered when interpreting the findings. First, seasonal variation in the underlying number of deliveries across the region was not accounted for, as our denominators reflect obstetric ICU admissions rather than the total population at risk. Without season-specific delivery denominators, we cannot calculate true population incidence, nor can we definitively rule out the possibility that regional delivery rates or peripheral hospital referral thresholds fluctuate during winter months.

Second, and most critically, the harsh winter weather in the Kashmir Valley introduces a major confounder that directly restricted our statistical approach. Heavy snowfall frequently causes severe road blockages and delayed emergency transfers for the large proportion of our cohort (56.63%) referred from peripheral facilities. Because the exact duration of these transport delays could not be reliably extracted from retrospective records, we opted not to perform multivariable logistic regression to avoid overfitting a model with unmeasured critical confounders. Consequently, our unadjusted analysis cannot statistically isolate the direct physiological effects of cold weather-such as cold-induced increases in left ventricular afterload-from the severe clinical deterioration caused by prolonged cardiogenic shock during a delayed presentation.

Third, because exact symptom onset dates were inconsistently documented before referral, ICU admission date was utilized as a proxy for seasonal classification. Due to the aforementioned winter transfer delays, a patient admitted in early winter may have developed symptoms in late autumn, potentially leading to misclassification of the seasonal exposure.

Finally, the retrospective chart review limited our ability to control for granular baseline differences, such as the exact severity of pre-existing gestational hypertension or baseline anemia, which may independently confound the observed unadjusted associations. Prospective, multicenter studies utilizing delivery-based denominators and tracking precise symptom-to-admission times are warranted to disentangle these environmental and logistical covariates.

## Conclusions

This study demonstrates a clinically and statistically significant seasonal variation in the proportion of obstetric ICU admissions attributable to PPCM, with the greatest burden concentrated in winter. These findings suggest a need to evaluate whether increased antenatal surveillance and a lower threshold for echocardiography in winter improve outcomes. Further prospective, multicenter investigation is needed to clarify the pathophysiological mechanisms that underlie this seasonal pattern and to inform season-specific preventive strategies.
